# Hospital Meetings, &c

**Published:** 1897-02-06

**Authors:** 


### HOSPITAL MEETINGS, &C.

### UNIVERSITY COLLEGE HOSPITAL.

A well-attended public meeting in aid of University College Hospital was held at the Drill Hall of the 19th Middlesex Rifles, Chenies Street, Gower Street, W.C., on Thursday, January 28th, to call attention to the serious financial condition of the hospital. The Duke of BEDFORD presided, and was supported on the platform by, amongst others, Lord Reay, G.C.S.I., Lord Monkswell, Sir Henry Thompson, the Right Hon. H. H. Asquith, Q.C., M.P., Mr. Christopher Heath, the Chief Rabbi, Captain H. M. Jessel, M.P., and Sir John Hutton.

The CHAIRMAN, after giving a very brief account of the growth of the hospital, said that while the work done by the hospital increased year by year the income had fallen off, with the result that the only courses now left open were either to curtail the expenditure or to increase the income. London, he pointed out, was about the only one of the large capitals which denied municipal aid to its hospitals. Paris gave £358,000 a year to hospitals, or 3s. 6d. per inhabitant, while the hospitals of Berlin, Vienna, Copenhagen, and Christiana were all rate-aided. He appealed to North Londoners to come to the rescue of the hospital, as he was sure that when they knew its need they would rally round it, their neglect proceeding not from want of heart but from want of thought. In conclusion, he moved a resolution declaring the hospital to be "a necessity for the well-being of the poor of St. Pancras and surrounding neighbourhoods."

The Right Hon. H. H. ASQUITH, whose rising was greeted with loud applause, in seconding, regretted the absence of the Bishop of London, who was to have moved the resolution, as he felt sure that nothing could have been more congenial to him than to make one of his earliest public appearances since he accepted that office in advocating an institution which appealed from the broad platform of humanity to men of all parties and all creeds. "The Door is Open" was the motto of University College Hospital, and that was the principle on which it had been and was being worked. It would be nothing short of a scandal if the hospital upon which that vast, crowded quarter of St. Pancras largely depended for the alleviation of the suffering of its poor and its sick were to be curtailed simply for want of public support, which, he felt confident, would be given if public intelligence and public knowledge could be brought to bear upon the case. This was an occasion on which a plain statement of fact and duty ought to be sufficient to bring home to everyone the duty under which they individually lay of giving such help as they could to the institution themselves, and, still further, the duty of making known to others the necessities of the hospital, the serious danger which threatened its future utility and beneficence, and, therefore, the grave obligation which lay on all those who had at heart the physical welfare of the poor and suffering of London, to assist, by doing everything in their power, to place the institution on a sound and permanent pecuniary foundation. (Loud applause.)

The resolution was carried.

The CHIEF RABBI, in moving the following resolution—

"That the North London or University College Hospital has proved during the sixty-three years of its existence of inestimable value to the sick poor, having treated during that period over a million patients, and ministering to the ailments of over 40,000 sick and injured persons annually,"

said that any little success in life to which he might have attained was eminently due to the training he had received at his *alma mater*, University College, where he had spent so many pleasant years. He appealed to the large number of his own co-religionists who had been educated both at the School and the College, and also to the great body of Nonconformists, to whom in those days before tests were abolished, University College opened wide and hospitably its doors, to come forward liberally to assist and support that hospital in the hour of its sore need. (Applause.)

Lord REAY, G.C.S.I., seconded, and the resolution was carried.

The Rev. HENRY LUKE PAGET proposed—

"That the North London or University College Hospital, now most urgently in need of funds, eminently deserves largely increased pecuniary support to enable it to carry on its humane duty of healing the sick, and to prevent the serious suffering and inconvenience which would be caused to the sick poor if its work were hampered and curtailed by the proposed closing of fifty beds on March 1st next."

Most of those who knew the neighbourhood in which the hospital stood knew that Euston Road masked and covered with the white fronts of its houses a mass of misery to which London could hardly produce a parallel. He had worked for a long time in the East-end, but had never seen anything to touch the awful misery of some parts of Somers Town. At the same time he was perfectly well persuaded that there was sufficient wealth in the district to which the hospital ministered to support a hospital of twice the size of University College.

Captain H. M. JESSEL, M.P., seconded the resolution, which was carried.

Sir JOHN HUTTON proposed, and Mr. CHRISTOPHER HEATH seconded, the following resolution, which was carried unanimously:—

"That this meeting pledges itself to use every effort—(*a*) To secure donations to clear off the debt amounting to £15,000, with which the institution is at present hampered; and (*b*) to place the charity on a more secure financial basis by the establishment of a 'Five Years Maintenance Fund.'"

A vote of thanks was accorded to the Chairman, on the motion of Lord MONKSWELL, seconded by Mr. HARRY LUCAS (the chairman of the Hospital Committee). The latter said that the neglect of its neighbours to support the hospital was well shown by the fact that the district it covered—one extending from New Oxford Street and Holborn on the south to Highgate on the north—only produced 351 annual subscribers. There were 12 subscribers in Tottenham Court Road and three in Gower Street.

The meeting then broke up.

### ROYAL LONDON OPHTHALMIC HOSPITAL.

A meeting in aid of the fund for the rebuilding of the Royal London Ophthalmic Hospital, in connection with which the foundation stone is to be laid by H.R.H. the Prince of Wales, was held at the Mansion House on Monday, the 1st inst., the LORD MAYOR in the chair.

The CHAIRMAN said that the position of the hospital at the present time was a peculiarly dangerous one, as having no room to provide for their cases in the old building they had commenced to build the necessary accommodation on a new site, and now lacked the funds to carry that work through.

Lord THRING, K.C.B., in moving a resolution placing on record the importance and usefulness of the hospital, made a heartfelt appeal (as one who had practically experienced the terrors of blindness, but who, through the aid of science, had been restored to sight) to all to extend to the poor the benefits which without an ophthalmic hospital would only be within the reach of the rich. In the year 1895 28,000 persons were treated at the hospital, of whom 800—and in those cases he took the greatest interest as he himself had suffered from the same disease—had been treated for cataract. That meant that during the last ten years 8,000 persons had been brought from darkness into light; it meant that hundreds of artisans, hundreds of clerks, and hundreds of helpless women were restored to their work. However much the sum asked for (£85,000) might seem, the benefits conferred were greater. (Applause.)

Sir DONALD CURRIE seconded, and read an extract from a letter which had been received from Mr. Gladstone, in which he said :—

"I find myself disabled by circumstances from giving any further attendance at public meetings. That need not, however, altogether prevent me from bearing my testimony to the value of institutions such as the Ophthalmic Hospital, in which the resources of science are made available to repair or mitigate the wastes of time, the defects of nature, or the deficiencies of disease. All who have suffered, even in the mildest form, from any of these causes acquire an enhanced perception of the immense value of the power of sight, and a new insight (in some degree) into the marvellous skill with which it is adapted to what I may call an infinity of purposes. Of those to whom you make your appeal a portion probably are already sufferers, but a larger number, now in the full enjoyment of their visual powers, must lay their account with having in the course of nature to stand hereafter in the same predicament. It will be no unhappy circumstance if, without waiting for the evil day, they stretch out the hand of help to their brethren."

The Rev. Brooke Lambert supported the resolution, which was carried.

The Right Hon. A. J. Mundella, M.P., moved a resolution of hearty appreciation of the hospital as a school of ophthalmic medicine and surgery. Very few, he thought, realised what suffering, what incapacity, what disability ophthalmia entailed, and how many, through its recurrence during after life, broke down and were prevented from earning their living. In connection with the enquiry into the condition of the Metropolitan Poor Law Schools, it had been found that in 1894, 8 per cent. of the children in those schools were actually under treatment for ophthalmia, while during their investigations on the Blind Commission, of which he was a member, they found that ophthalmia was the prevailing cause of blindness. He therefore urged on all persons, now they had before them an institution which had done more to repress and to stamp out that disease than any other institution in the kingdom, asking for their help, to come liberally to its aid.

Mr. Jonathan Hutchinson, F.R.S., seconded, and said that the prevention of blindness in all its various forms was an economic matter of the greatest national importance. In support of that he had calculated in two instances the financial saving to the public of a hospital such as the Royal London Ophthalmic Hospital. He first took the disease known as glaucoma, one which in its acute form attacked healthy persons, and if not at once treated caused irremediable blindness. Assuming the wage earned to be £1 a week, and the age at which persons were attacked by this disease to be 45, a period when they might have looked to a further ten to fifteen years of useful life, during which they would have continued to earn their own livelihood, and to have been an advantage instead of an incumbrance to the community, he calculated that as about twenty operations a year for this disease were performed at the hospital, the pecuniary gain to the community was something like £12,000. But that was one form of eye disease only, and numerically a very small one. As to cataract, a very much more common disease, about 400 cases of which were operated on during the year at the Royal London Ophthalmic Hospital, assuming that only half the above-mentioned period of wage-earning life remained to the patients from that form of disease, he estimated that the gain to the community from that one hospital alone was something like £128,000 a year. The Royal London Ophthalmic Hospital stood at the very centre of ophthalmic science, and it was one of the very fountain heads from which the knowledge of the treatment of eye diseases extended.

Mr. John Tweedy supported, and said that it had been stated that the work done at the special hospitals could be as well done in the special departments of the general hospitals. He agreed that that was true with respect to many special departments, but he unhesitatingly said, after a very intimate knowledge extending over a quarter of a century of a special department at a general hospital, and of the work done at the Moorfields Hospital, that it would be impossible to do, or to teach, at any general hospital the work which they did and taught at Moorfields.

The resolution was put, and carried unanimously.

Cardinal Vaughan moved, the Chief Rabbi seconded, and Messrs. C. Ridley Smith and Percy Thornton, M.P. supported the following resolution, which was carried unanimously :—

"That this meeting approves of the rebuilding the hospital on the site chosen in the City Road, recognises the urgent necessity of its speedy completion, and promises by every effort in its power to secure liberal aid from all who sympathise with ophthalmic suffering."

A vote of thanks to the Chairman having been moved,

The Lord Mayor, in responding, suggested that great efforts should be made by the promoters of this fund for rebuilding to get annual subscribers. £85,000, he said, was a large sum to raise with one effort, especially at the present time; while £5,000 a year, raised in annual subscriptions, would in about 20 years repay the loan of the sum mentioned.

The proceedings then terminated.

